# Research Progress of Composite Films in Postharvest Preservation of Fruits and Vegetables

**DOI:** 10.3390/molecules31111968

**Published:** 2026-06-05

**Authors:** Yiru Zhu, Danni Li, Junzhe Qu, Hongliang Zhu, Liqun Ma

**Affiliations:** 1 Sichuan Advanced Agricultural and Industrial Institute, China Agricultural University, No. 515 Xingyuan 8th Road, Xinjin District, Chengdu 611430, China; zhuyr39@126.com; 2 The College of Food Science and Nutritional Engineering, China Agricultural University, Beijing 100083, China; 15137992773@163.com (D.L.); 13653755057@163.com (J.Q.); hlzhu@cau.edu.cn (H.Z.)

**Keywords:** postharvest preservation of fruits and vegetables, composite films, active packaging, mechanism of action, precision application

## Abstract

Fruits and vegetables are vulnerable to enhanced respiratory metabolism, water loss, enzymatic browning, and microbial invasion during postharvest storage and transportation, leading to spoilage and reduced shelf life. Traditional preservation methods, such as physical, chemical, and single-component film technologies, offer limited benefits due to their single function and instability. Composite films, however, show great promise in postharvest preservation by integrating the strengths of different substrates and active components. This review discusses the research progress of composite films in preserving fruits and vegetables, covering their definition, classification, and key performance characteristics. It also examines the functional roles of substrates like polysaccharides, proteins, and lipids, and explores the mechanisms of composite films, including gas regulation, metabolic inhibition, water retention, antimicrobial effects, antioxidant activity, and browning delay. Additionally, it compares the applications of composite films in whole and fresh-cut fruits and vegetables. Current research highlights the shift from general applications to targeted designs based on the unique deterioration mechanisms of different produce. Future studies should focus on optimizing the matching between product characteristics and film design, advancing composite films towards safer, more stable, and practical applications.

## 1. Introduction

Fresh fruits and vegetables are an indispensable part of the human diet. They are rich in vitamins, minerals, dietary fiber, and various antioxidant compounds [1]. However, fruit and vegetable tissues are usually tender and juicy, have high water content, and have relatively weak surface protective layers. Therefore, they still maintain strong biological activity after harvest and are easily affected by water transpiration, respiratory metabolism, enzymatic reactions, and microbial invasion during storage, transportation, and sales. These changes can lead to quality deterioration, shortened shelf life, and reduced commercial value [2,3]. Postharvest deterioration of fruits and vegetables is a dynamic process jointly driven by multiple physiological and biochemical processes. Its main manifestations usually include enhanced respiratory metabolism, water transpiration, enzymatic browning, and microbial invasion. These processes interact with each other and eventually cause decay, nutrient loss, and sensory quality decline [4]. Overall, postharvest deterioration of fruits and vegetables is not caused by a single factor, but by the combined effects of respiratory metabolism, water migration, enzymatic reactions, and microbial proliferation. Therefore, effective postharvest preservation technologies should target these key processes to achieve more stable quality maintenance [1,4,5].

Traditional fruit and vegetable preservation technologies mainly include physical preservation, chemical preservation, and single-component film preservation. These methods have played an important role in extending shelf life, but each has its own application boundaries and limitations. Therefore, they are often unable to independently meet the diverse preservation needs of different fruits and vegetables during commercial distribution [5,6]. Physical preservation technologies mainly delay fruit and vegetable senescence by regulating the storage environment or applying physical treatments. Low-temperature storage is one of the most common methods. It slows the respiration rate of fruits and vegetables, delays physiological senescence, and inhibits microbial growth by reducing temperature, thereby extending storage life. However, low-temperature treatment is not suitable for all fruits and vegetables. Some products are prone to chilling injury under low-temperature conditions. In addition, cold-chain operation is costly and highly dependent on energy and infrastructure, so its use is limited in some regions and for some product categories [1,5,6]. Controlled-atmosphere preservation suppresses respiration and slows ripening by regulating the proportions of oxygen and carbon dioxide in the storage environment. It has been widely used for fruits such as apples and bananas during long-distance transportation. However, this technology has strict requirements for environmental gas composition. If control is improper, it may induce anaerobic metabolism, aggravate decay, or cause abnormal ripening. Therefore, it requires high levels of precise control and equipment support [7]. Physical methods such as ultraviolet irradiation, ultrasound, and ozone treatment have also been widely studied in recent years. UV-C can delay fruit and vegetable decay by inhibiting pathogenic microorganisms, but excessive irradiation may cause tissue damage and nutrient loss [4,8]. Ultrasound treatment helps reduce pesticide residues, inhibit enzyme activity, and decrease microbial contamination, but excessive treatment may also damage tissue texture [5,9]. As a strong oxidant, ozone can kill microorganisms and reduce ethylene concentration, thereby delaying ripening. However, its concentration must be strictly controlled, because excessive ozone may cause oxidative damage to fruit and vegetable surfaces [9,10]. This indicates that although physical preservation methods introduce fewer chemical residues, they often still have problems such as limited applicability and sensitivity to treatment conditions in practical applications [6].

Chemical preservation technologies mainly delay fruit and vegetable senescence and decay through the use of preservatives, ethylene regulators, plant essential oils, and ethylene scavengers. Among them, ethylene regulators such as 1-MCP can slow fruit and vegetable ripening by inhibiting ethylene receptor activity, but improper treatment conditions may also affect flavor and taste. Plant essential oils, such as tea tree oil, thyme oil, and cinnamon oil, have attracted extensive attention in fruit and vegetable preservation because of their antimicrobial, antioxidant, and antifungal activities. However, the concentration and application method of essential oils must be strictly controlled, because excessive use may alter the odor and color of fruits and vegetables [11,12]. In addition, ethylene scavengers such as TiO_2_ and KMnO_4_ can delay ripening by oxidizing ethylene, but their effectiveness decreases over time, and some materials may also affect the stability of the packaging system [13]. Therefore, although chemical preservation technologies have certain advantages, they still face problems related to residue-related safety concerns, environmental impact, and limited adaptability.

Single-component film-based preservation mainly relies on forming a physical barrier on the surface of fruits and vegetables or around the product to reduce water evaporation and regulate gas exchange [8], thereby slowing respiration and extending shelf life. Common materials include polyethylene films, wax films, polyvinyl alcohol films, and cellulose films [10]. Some of these film materials are derived from natural biopolymers and have good safety and biodegradability [14,15]. However, single-component films usually have problems such as insufficient gas permeability, limited mechanical properties, easy breakage, and possible negative effects on the sensory quality of fruits and vegetables [6,16]. For example, some film materials have poor oxygen permeability, which can easily create a low-oxygen environment inside the package [8]. This may affect normal fruit and vegetable ripening or induce anaerobic metabolism. In addition, surface coatings such as wax films may change the texture and appearance of fruits and vegetables in some cases. In summary, traditional physical, chemical, and single-component film preservation technologies play a certain role in delaying postharvest deterioration of fruits and vegetables. However, they still have limitations such as limited applicability, single functionality, and insufficient stability [16]. Therefore, these technologies are difficult to use alone to meet multiple preservation needs, including water retention, gas regulation, antimicrobial protection, and quality maintenance. In this context, composite films have attracted considerable attention in postharvest preservation of fruits and vegetables because they can integrate the advantages of different materials and functional components [6]. However, many existing studies mainly emphasize the positive effects of composite films, while the differences among material systems, the reasons for inconsistent preservation performance, and the matching relationship between film properties and produce-specific deterioration mechanisms remain insufficiently discussed. Therefore, this review goes beyond a descriptive summary of composite films in postharvest preservation. Instead, it critically compares the advantages, limitations, and application boundaries of different composite film systems. Particular attention is paid to the structure–property–preservation relationship, the trade-offs among mechanical, barrier, and biological properties, and the future development of produce-specific film design.

For transparency, this narrative review was based on a broad literature search of peer-reviewed articles related to composite films, composite coatings, active packaging, and postharvest preservation of fruits and vegetables. The literature was mainly retrieved from Web of Science, ScienceDirect, SpringerLink, Wiley Online Library, and Google Scholar. The search terms included “composite film”, “composite coating”, “edible film”, “edible coating”, “active packaging”, “bio-based film”, “nanocomposite film”, “postharvest preservation”, “fruit”, “vegetable”, “fresh-cut produce”, “controlled release”, and “intelligent packaging”. The search mainly covered publications from 2015 to 2026, with earlier representative studies included when they provided fundamental concepts, classical mechanisms, or widely used evaluation methods. Studies were included if they focused on composite films or coatings for fruit and vegetable preservation and reported material composition, functional properties, mechanisms, or application effects. Studies unrelated to postharvest fruits and vegetables, non-food packaging materials, duplicated publications, or papers lacking sufficient information on film composition or preservation effects were excluded [17].

## 2. Concepts, Classification, and Performance Characteristics of Composite Films

### 2.1. Definition and Concept of Composite Films

Composite films are film materials formed by combining two or more materials through physical or chemical methods. Their design goal is to overcome the limitations of single-component films in gas permeability, mechanical strength, moisture resistance, and preservation functions, and to achieve multiple performance improvements through synergistic interactions among different materials. In the field of food packaging, composite films are usually constructed by combining natural polymers and synthetic polymers, or by combining different natural polymers, to obtain film structures that are more suitable for postharvest preservation of fruits and vegetables [18].

It should be noted that “films” and “coatings” are closely related but not identical concepts [19]. Composite films are usually pre-formed and self-supporting materials that are prepared before being applied to fruits, vegetables, or packaging systems. They are often evaluated as independent packaging materials in terms of thickness, tensile strength, elongation, gas permeability, and water vapor transmission [19,20]. In contrast, composite coatings are usually formed directly on the surface of fruits and vegetables by dipping, spraying, brushing, or layer-by-layer deposition. Their performance is more strongly affected by surface adhesion, coating uniformity, drying behavior, produce morphology, and interaction with the epidermal structure [21,22]. Therefore, films and coatings differ in preparation method, structural behavior, application mode, and industrial implementation. In this review, the term “composite films” is used as a broad term when discussing material systems and mechanisms, while “composite coatings” is used when the cited studies specifically involve direct surface coating treatments.

In addition to preparation and application mode, thickness is also an important criterion for distinguishing films from other sheet-like or laminate materials. In polymer and packaging contexts, films are generally regarded as thin, continuous, and flexible materials, and a practical thickness boundary of approximately 200 μm is often used to distinguish films from thicker sheets or plate-like materials [23]. Therefore, in this review, “composite films” mainly refer to thin composite materials with a thickness generally not exceeding 200 μm. When the thickness is greater than 200 μm, especially in multilayer or highly rigid structures, the material is more appropriately described as a composite sheet, laminate, or plate rather than a film [23]. This distinction is important because thickness can significantly affect gas and water vapor transmission, mechanical strength, flexibility, transparency, drying behavior, and final preservation performance [24].

Compared with traditional single-component films, the most prominent feature of composite films is their structural and functional design flexibility. Studies have shown that composite films can combine barrier properties, mechanical strength, antimicrobial activity, and antioxidant activity in the same system through the rational matching of different materials. For example, when antimicrobial agents, antioxidants, or nanoparticles are introduced onto the film surface or into the film matrix, microbial invasion and oxidative damage can be more effectively inhibited. These features give composite films clear advantages in extending the shelf life of fruits and vegetables and maintaining their quality. The value of composite films in postharvest preservation of fruits and vegetables is not only reflected in shelf-life extension, but also in the greater design space they provide for green packaging materials and functional food packaging [6,25,26].

### 2.2. Classification of Composite Films

**Figure 1:**
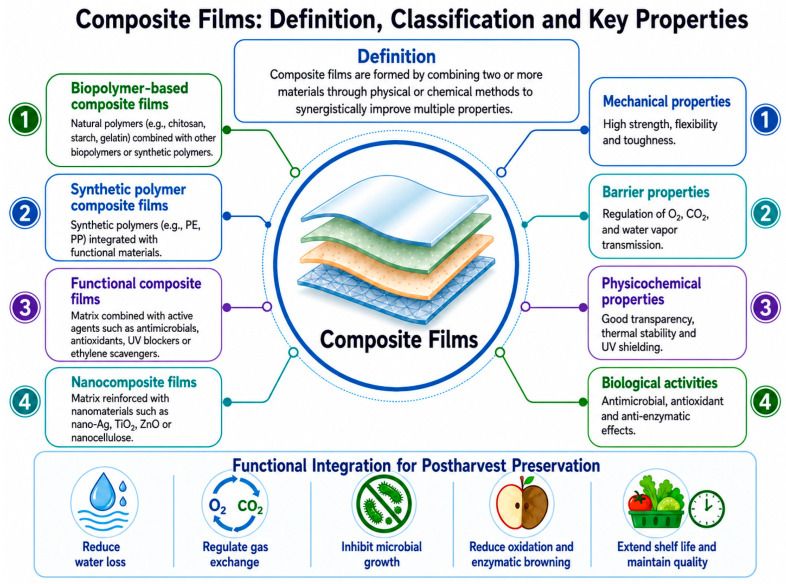
Definition, classification, and key performance characteristics of composite films.

From the perspectives of material source, matrix structure, functional design, and reinforcement strategy, composite films can be described using several classification dimensions, including bio-based composite films, synthetic composite films, functional composite films, and nanocomposite films [27,28]. These terms are used in this review as descriptive categories with different design emphases, rather than as strictly independent or mutually exclusive groups. The main classification dimensions of composite films are summarized in Figure 1.

Bio-based composite films are usually prepared by combining natural polymers such as chitosan, gelatin, alginate, and soy protein with other bio-based materials or a small amount of synthetic materials [29]. The main advantages of these film materials are biodegradability, environmental friendliness, and good biocompatibility. Some substrates also have inherent antimicrobial and antioxidant abilities. For example, chitosan/polyvinyl alcohol composite films are considered to have good antimicrobial properties and can delay fruit and vegetable decay to a certain extent [27]. However, the moisture-barrier properties and mechanical strength of bio-based composite films often still require further optimization [30]. Synthetic composite films are mainly composed of synthetic polymers such as polyethylene, polypropylene, and polyvinyl chloride combined with other functional materials. Their advantages include stable physical properties, heat resistance, moisture resistance, and relatively high mechanical strength. Therefore, they still occupy an important position in long-term storage and transportation packaging [29]. However, their major problem is poor degradability, which may cause environmental burdens. For this reason, many recent studies have attempted to combine degradable materials with synthetic materials in order to balance performance and environmental friendliness [28]. Functional composite films are film systems formed by introducing antimicrobial agents, antioxidants, ultraviolet-shielding agents, or active-release components into basic film structures. In addition to general barrier functions, these films can improve fruit and vegetable preservation by actively intervening in oxidation reactions, microbial growth, and ripening and senescence. For example, tea tree oil/chitosan composite films can not only inhibit bacterial growth, but also reduce water evaporation and improve antioxidant activity. They therefore have good application prospects in fruit and vegetable packaging systems with high requirements for safety and active preservation functions [31]. Nanocomposite films are systems in which nanosilver, nano-TiO_2_, nano-alumina, or other nanomaterials are introduced to enhance film performance. Because nanomaterials have large specific surface areas and special physicochemical properties, they can often significantly improve antimicrobial activity, antioxidant activity, gas-barrier properties, and mechanical strength [27]. For example, nanosilver/polymer composite films show clear advantages in extending the preservation period of fruits and vegetables and improving antimicrobial activity [28,31]. Overall, different types of composite films have different emphases in raw-material sources, structural characteristics, and functional positioning. Bio-based composite films emphasize environmental friendliness and safety; synthetic composite films emphasize stability and durability; functional composite films emphasize active intervention; and nanocomposite films emphasize nanoscale reinforcement and performance enhancement. This classification also provides a basic framework for subsequent formulation design and application selection of composite films [27,28]. It should be emphasized that the above categories should not be understood as rigid and mutually exclusive taxonomic groups. In practical formulation design, one composite film may simultaneously belong to more than one descriptive category. For example, a chitosan-based film incorporated with nano-Ag, nano-TiO_2_, ZnO, or plant essential oils can be regarded as a bio-based or bio-derived composite film because of its polymer matrix, a functional composite film because of its antimicrobial or antioxidant activity, and a nanocomposite film when nanoparticles are introduced as reinforcing or active components [27,28,31]. Therefore, the classification used in this review is intended to clarify different design emphases rather than to establish isolated material classes. Bio-based and synthetic composite films mainly reflect material origin and matrix composition; functional composite films highlight active preservation functions; and nanocomposite films emphasize nanoscale reinforcement, carrier effects, or performance enhancement. This clarification helps avoid conceptual redundancy and provides a more logical basis for subsequent discussions on formulation design, performance evaluation, and application selection.

### 2.3. Main Performance Characteristics of Composite Films

The application value of composite films in postharvest preservation of fruits and vegetables mainly depends on whether their mechanical properties, barrier properties, physicochemical properties, and biological activities can be synergistically improved [32]. Existing studies generally indicate that these four types of properties correspond to film integrity, mass-transfer control, structural stability, and active intervention against oxidative and microbial deterioration, respectively [30]. Therefore, evaluation should not focus on only one indicator, but should consider the overall preservation effect.

Mechanical properties are the basis for composite films to play a protective role in practical packaging. They mainly include tensile strength, tear resistance, and flexibility. Studies have shown that the tensile strength of alginate/arabinoxylan composite films can reach 42.5 MPa [26]. When titanium dioxide nanoparticles are added, the mechanical strength of the composite films can be further increased by about 25%. This suggests that structural optimization and nano-enhancement can significantly improve the mechanical properties of films.

To provide a more practical context, this value should be interpreted in comparison with commonly used commercial packaging films. The tensile strength of many LDPE film-grade materials is generally reported within a lower or comparable range, whereas some HDPE films and PP films show similar or moderately higher mechanical strength. In contrast, oriented PET films usually exhibit much higher tensile strength. Therefore, a tensile strength of 42.5 MPa indicates that alginate/arabinoxylan composite films may already approach the mechanical level required for some flexible packaging applications, although direct comparison should be made cautiously because tensile strength is affected by film thickness, testing direction, humidity, plasticizer content, and testing method [33]. Thus, mechanical strength should be evaluated together with elongation at break, puncture resistance, water vapor transmission rate, oxygen transmission rate, and actual preservation performance.

Barrier properties are one of the key factors in extending fruit and vegetable shelf life. They mainly include the regulation of oxygen, carbon dioxide, and water vapor. Studies have shown that the oxygen transmission rate of polyethylene/cellulose composite films is about 50% lower than that of single polyethylene films [34], and the water vapor transmission rate is reduced by about 40%, thereby reducing water loss and oxidative reactions in fruits and vegetables. In addition, the water vapor transmission rate of chitosan–lignin composite films is clearly lower than that of traditional polyethylene films [35], showing good moisture-barrier performance. Therefore, improvement in the barrier properties of composite films is often directly related to their preservation performance.

Physicochemical properties mainly include transparency, thermal stability, and water solubility. These indicators directly affect the packaging performance and environmental adaptability of films. Studies have found that polyethylene/lignin composite films can have transparency above 80% [36] and certain ultraviolet-shielding ability, which helps reduce the effect of light exposure on fruit and vegetable quality [37]. In addition, alginate–arabinoxylan composite films can maintain good structural stability at relatively high temperatures [38], indicating that some composite films have good thermal stability and application adaptability [39].

Biological activity is one of the important features that distinguishes composite films from ordinary packaging films. It usually includes antimicrobial and antioxidant activities. Studies have shown that chitosan composite films containing thyme essential oil have strong inhibitory effects against *Escherichia coli and Staphylococcus aureus* [36], while lignin–curcumin composite films show a high DPPH free-radical scavenging rate and can effectively delay oxidative deterioration of fruits and vegetables [15].

The optimization of composite films is usually accompanied by trade-offs among different properties. For example, improving oxygen-barrier properties may suppress respiration and delay ripening, but excessive gas restriction may induce anaerobic metabolism and off-flavor formation. Improving water vapor barrier properties can reduce weight loss, but excessive humidity inside the package may promote condensation and microbial growth. Incorporating essential oils or plant extracts can improve antimicrobial and antioxidant activities, but high concentrations may affect the odor, color, and sensory acceptance of fruits and vegetables [40,41]. Similarly, nanomaterials can enhance mechanical and barrier properties, but their migration behavior and safety risks remain important concerns [42,43].

These examples indicate that the performance optimization of composite films should not focus only on maximizing a single material property. Instead, quantitative indicators such as tensile strength, elongation at break, oxygen transmission rate, water vapor transmission rate, antimicrobial activity, and antioxidant activity should be interpreted together with actual preservation outcomes, including weight loss, firmness, browning degree, microbial load, nutrient retention, and sensory quality [32,44,45]. This is particularly important because a film with superior mechanical or barrier properties may not necessarily show the best preservation performance if its mass-transfer behavior does not match the physiological characteristics and dominant deterioration mechanisms of the target produce. Therefore, a more meaningful evaluation should connect film structure, mass-transfer behavior, active-component release, sensory quality, and produce-specific preservation outcomes.

### 2.4. Application Advantages of Composite Films in Postharvest Preservation of Fruits and Vegetables

Compared with single-component film materials, the advantages of composite films in postharvest preservation of fruits and vegetables are mainly reflected in their ability to simultaneously exert multiple effects, including physical barrier protection, gas regulation, antioxidant activity, and antimicrobial activity through multi-component synergy [38]. This provides more comprehensive and stable quality protection for fruits and vegetables [19].

First, composite films can reduce water loss by forming a denser surface barrier and regulate the gas composition inside the package [45], thereby delaying postharvest ripening. Studies have shown that when PLA/PBAT composite films were used for passion fruit packaging, weight loss, shriveling index, and firmness were well maintained after storage at 20 °C for 21 days, while the increase in ethanol content was also inhibited. This indicates good gas-regulation capacity. Similarly, chitosan–pectin composite coatings can delay the respiratory peak of mangoes and maintain better firmness and titratable acidity [20], thereby delaying fruit senescence. This suggests that the advantages of composite films are first reflected in their ability to achieve both physical barrier protection and metabolic regulation.

Second, composite films can significantly enhance antioxidant and antimicrobial properties by introducing natural active components. Studies have found that sodium alginate/pectin composite films containing selenium nanoparticles not only have strong free-radical scavenging ability [46], but also effectively inhibit *Escherichia coli and Staphylococcus aureus*. In strawberry storage, they can delay decay and maintain total phenol and total flavonoid contents. In addition, protocatechualdehyde-crosslinked gelatin/sodium hyaluronate composite coatings show high DPPH radical scavenging activity and clear antimicrobial activity, extending the shelf life of strawberries from 4 days to 8 days [47]. These findings suggest that composite films can more effectively address postharvest oxidative damage and microbial invasion through functional activity enhancement.

Composite films can also improve mechanical and barrier properties through structural complementarity and interfacial synergy. Studies have shown that gelatin–chitosan composite films form a relatively dense network structure through hydrogen bonding, and their tensile strength and elongation at break are both better than those of single-component films. In addition, caseinate-carboxymethyl chitosan-soybean oil composite coatings can effectively reduce blueberry weight loss through hydrophobic modification by lipid components and inhibit yeast and mold growth [48]. This suggests that the advantages of composite films come not only from the introduction of active components, but also from structural complementarity and optimization among materials.

Finally, composite films also show greater potential for functional expansion. In recent years, some studies have introduced temperature-responsive components, cyclodextrin inclusion complexes, and plant extracts to construct composite film systems with intelligent response or on-demand release characteristics. These film materials not only improve the stability and utilization efficiency of hydrophobic active components [49], but also further extend the shelf life of berries, tomatoes, kiwifruit, and other products [50,51]. This indicates that the application advantages of composite films in postharvest preservation of fruits and vegetables have expanded from basic protective functions to precision regulation and multifunctional synergy, showing their comprehensive application potential in postharvest quality maintenance and functional preservation.

Overall, composite film technology can more efficiently maintain the freshness and nutritional quality of fruits and vegetables through the synergistic action of physical barriers, gas regulation, and functional active components [45]. It also shows clear advantages in extending shelf life, reducing postharvest losses, and promoting the application of green packaging materials [46]. Therefore, composite films have become an important material system with both theoretical research value and application prospects in postharvest preservation of fruits and vegetables [38].

## 3. Composition Systems and Functional Construction of Composite Films

### 3.1. Main Substrates and Functional Characteristics

Polysaccharides, proteins, and lipids are the main substrate types used in composite films for postharvest preservation of fruits and vegetables. Different substrates provide different film-forming properties, mechanical strength, barrier performance, biodegradability, and active-component loading capacity. Therefore, substrate selection is a key step in the design of composite films. The main substrate types and their functional characteristics are summarized in Table 1.

#### 3.1.1. Polysaccharide-Based Substrates

Polysaccharide materials are among the most widely used basic substrates in composite films for postharvest preservation of fruits and vegetables. They mainly include chitosan, starch, alginate, and pectin [52]. These materials are widely available and generally have good biodegradability and strong film-forming ability. Therefore, they are often used as the main structural matrices of composite films [20]. Among them, chitosan has both good film-forming ability and certain natural antimicrobial activity, so it is especially widely used in research on fruit and vegetable preservation films. Alginate and pectin are more suitable for constructing edible coating systems with good uniformity because they have relatively high film-forming stability and certain gas-barrier performance [52]. In contrast, starch is abundant and low-cost, but when used alone to form films, it often has problems such as high moisture absorption, insufficient water resistance, and weak moisture-barrier properties. Therefore, it usually needs to be combined with other materials [20]. Overall, polysaccharide-based substrates mainly serve as film-forming matrices, gas-barrier structures, and active-component carriers in composite films.

#### 3.1.2. Protein-Based Substrates

Protein-based substrates mainly include gelatin, whey protein, and soy protein [21]. Compared with polysaccharide materials, protein-based films usually have better film-forming ability, transparency, and flexibility, and they are more likely to form continuous and uniform film layers [54]. At the same time, protein molecules contain many functional groups that can interact with active compounds. Therefore, protein-based films are usually more suitable as loading matrices for functional components such as antioxidants, antimicrobial agents, and natural extracts [21]. Studies have shown that when gelatin is combined with polysaccharides such as sodium alginate or pectin, the gas-barrier properties, mechanical strength, and water resistance of the films can be further improved [53]. These improvements help delay postharvest ripening and oxidative changes in fruits and vegetables. However, protein-based films generally have high hydrophilicity and insufficient moisture resistance. Therefore, in practical applications, they often need to be combined with polysaccharides, lipids, or cross-linking agents to improve their comprehensive performance [54].

#### 3.1.3. Lipid-Based Substrates

Lipid materials mainly include plant oils, waxes, and fatty acids. Their core role in composite films is to improve film hydrophobicity and moisture-barrier performance. Compared with polysaccharides and proteins, lipid materials have stronger water vapor barrier properties. Therefore, they are often used in postharvest fruit and vegetable preservation to reduce water loss caused by transpiration and delay surface shriveling. For example, natural waxes such as beeswax and carnauba wax can be used to coat the surfaces of fruits and vegetables to reduce water loss and decay [55]. However, when lipid materials are used alone to form films, they usually have problems such as high brittleness, weak mechanical properties, and insufficient film continuity. Therefore, they are more suitable for constructing composite systems together with polysaccharide or protein materials [53]. Thus, lipid materials mainly function as hydrophobic modification components in composite film systems. Their role is more focused on improving the water-barrier properties of films rather than serving as the main film-forming materials alone [55].

#### 3.1.4. Cellulose-Derived Reinforcing Systems

Cellulose-derived materials are often discussed separately because they are more commonly used as reinforcing phases rather than as primary film-forming substrates in composite films. They mainly include cellulose nanocrystals, cellulose nanofibers, and nanocellulose [56]. These materials have high crystallinity and large specific surface areas, and can improve the mechanical properties, thermal stability, and barrier properties of films through filling effects and interfacial interactions [52]. Studies have shown that when cellulose nanomaterials are combined with polysaccharide or protein matrices, they usually help form denser film structures and reduce water vapor transmission rate, thereby improving the application stability of fruit and vegetable preservation films [53]. Therefore, in composite film systems, cellulose-based materials are usually not used as the main film-forming substrates. Instead, they are more often present as reinforcing phases, and their main role is to improve film structural stability, moisture-barrier properties, and overall application performance [56].

## 4. Functional Components and Their Action Characteristics

### 4.1. Natural Antioxidant Components

In composite films for postharvest preservation of fruits and vegetables, natural antioxidant components are among the most common functional ingredients. They mainly include tea polyphenols, proanthocyanidins, gallic acid, and other plant polyphenols [57]. These components usually have strong free-radical scavenging ability and can slow postharvest oxidative damage and enzymatic browning in fruits and vegetables. At the same time, hydroxyl groups in polyphenols can form hydrogen bonds with polysaccharide or protein matrices, thereby improving the film network structure and increasing film compactness and stability [58]. Taking tea polyphenols as an example, related studies show that when they are introduced into pectin-konjac glucomannan composite films, the antioxidant and antimicrobial properties of the films are significantly enhanced. At an appropriate addition level, the tensile strength and surface hydrophobicity of the films also increase [59]. However, the addition of phenolic components is not necessarily better at higher levels. When the addition level is too high, active compounds may aggregate in the film system, which is not conducive to maintaining film uniformity and comprehensive performance [59].

### 4.2. Natural Antimicrobial Components

Natural antimicrobial components mainly include plant essential oils and their active monomers, natural antimicrobial peptides, and plant-derived extracts [60]. Among them, essential oil components such as thymol, carvacrol, eugenol, and cinnamaldehyde are the most widely used in composite films because they usually have good antimicrobial and antioxidant potential [57]. Compared with direct addition to food systems, introducing essential oils into film matrices is more conducive to stabilization and sustained release, because film materials can reduce volatilization losses to some extent and prolong the action time [60]. However, the final activity of essential oil components does not depend only on their own properties. It is also closely related to matrix compatibility and release behavior [60]. Therefore, in composite film systems, the application focus of natural antimicrobial components is not merely the addition of essential oils. Rather, the key is to improve their stability and sustained antimicrobial ability through rational structural design [57].

However, the application of essential-oil-loaded composite films still faces an important sensory barrier. Most essential oils and their active monomers are highly volatile and have strong characteristic aromas. When the loading concentration is too high or the release rate is too rapid, these compounds may mask the natural aroma of fruits and vegetables, change their original flavor, or even produce an unacceptable odor, thereby reducing consumer acceptance [40,41]. This limitation is particularly important for fresh fruits, fresh-cut products, and minimally processed vegetables, because these products are usually consumed directly and consumers are highly sensitive to changes in natural flavor, aroma, and freshness perception [40]. Therefore, the practical value of essential-oil-loaded composite films should not be evaluated only by antimicrobial or antioxidant activity. A balance should be established among active-compound concentration, release rate, preservation efficacy, and sensory acceptability.

To overcome this limitation, encapsulation, nanoemulsification, cyclodextrin inclusion, multilayer film construction, and controlled-release systems have been proposed to reduce burst release and improve the stability of essential oils [40,41,61]. These strategies can help maintain antimicrobial activity while reducing excessive odor release during storage. Future studies should further combine microbiological evaluation with sensory analysis, volatile-compound monitoring, and consumer acceptance tests, so as to determine the effective concentration range that can achieve preservation effects without negatively affecting product flavor.

### 4.3. Natural Pigments and Indicator Components

Natural pigment components mainly include curcumin, anthocyanins, and carotenoids [62]. In addition to certain antioxidant and antimicrobial abilities, these components can also provide films with ultraviolet shielding, photoresponsive functions, or freshness-indicating functions. Therefore, they have potential for both active packaging and intelligent packaging [57]. Curcumin is a widely studied natural pigment. It has antioxidant, antimicrobial, and multiple biological activities, but it also has poor water solubility and insufficient stability. Therefore, its application in composite films often needs to be combined with encapsulation, emulsification, or delivery-system design to improve its dispersion and utilization efficiency [62]. Anthocyanins are often used to construct responsive composite films because their color is sensitive to pH changes, which can indicate changes in the quality state of fruits and vegetables during storage. Therefore, natural pigment components in composite films not only provide active functions, but also expand the possibility of developing composite films for intelligent packaging.

### 4.4. Nano-Reinforcement and Carrier Components

In addition to natural active components, nano-reinforcement and carrier components are also important in the current functional design of composite films. They mainly include ZnO, TiO_2_, SiO_2_, layered clay, metal–organic framework materials, and nanocellulose [57]. The roles of these components in composite films are mainly reflected in two aspects. On the one hand, they can improve the mechanical and barrier properties of films by strengthening interfacial interactions and extending the diffusion paths of water and gases. On the other hand, they can also serve as carriers that protect active components and enable the sustained release of antimicrobial agents, antioxidants, and volatile active compounds [63]. Therefore, nano-components in composite films are both reinforcing phases and important parts of functional delivery systems.

### 4.5. Nanoparticle Migration, Safety, and Regulatory Concerns

Although nanomaterials can improve the mechanical, barrier, antimicrobial, and controlled-release properties of composite films, their potential risks should be critically considered [42,43,51,56]. Nanoparticles such as nano-Ag, TiO_2_, ZnO, and other metal or metal-oxide nanomaterials may migrate from the film matrix into food matrices or food simulants under certain conditions, especially when the film is used under conditions of high humidity, acidic environments, prolonged storage, or direct-contact coating systems. The migration behavior is affected by nanoparticle size, surface charge, dispersion state, matrix compatibility, film degradation, storage temperature, and the chemical composition of fruits and vegetables [43,64].

Potential toxicological concerns include oxidative stress, cell membrane damage, bioaccumulation, and long-term exposure risks [42,43]. In addition, nanomaterials released during production, use, or disposal may also create environmental concerns. Therefore, the application of nanocomposite films should not be evaluated only by antimicrobial or barrier performance. Migration testing, cytotoxicity evaluation, dietary exposure assessment, environmental risk analysis, and compliance with food-contact material regulations are also necessary [43]. Future studies should give more attention to safe-by-design nanocomposite films, in which nanomaterials are immobilized within stable matrices or replaced by biodegradable and low-risk nano-reinforcement systems.

## 5. Evaluation Methods for Composite Films

### 5.1. Evaluation of Mechanical Properties

Mechanical properties are basic indicators used to assess the practical application potential of composite films. They usually include tensile strength, elongation at break, and Young’s modulus [65]. Tensile strength reflects the ability of a film to resist rupture under external force. Elongation at break characterizes the flexibility and extensibility of the film. Young’s modulus is used to evaluate the rigidity of the film material [66]. For postharvest preservation films for fruits and vegetables, higher tensile strength helps maintain packaging integrity, while appropriate elongation at break helps improve packaging adaptability and resistance to rupture. Therefore, mechanical-property evaluation is usually regarded as the primary criterion for film-forming quality and application feasibility of composite films [66].

### 5.2. Evaluation of Barrier and Surface Properties

Barrier properties are among the core indicators used to evaluate composite films for fruit and vegetable preservation. They mainly include water vapor transmission rate, oxygen transmission rate, and carbon dioxide transmission rate [67]. Among them, water vapor transmission rate is a key parameter for evaluating the moisture-barrier performance of films, because postharvest water loss is an important cause of quality deterioration in fruits and vegetables [68]. In addition to gas and water vapor barrier properties, contact angle, moisture absorption, swelling rate, and surface hydrophobicity are also often used to evaluate the response behavior of film surfaces to water. Together, these indicators can reflect the potential ability of the film layer to control water loss caused by transpiration and regulate the packaging microenvironment of fruits and vegetables [67].

### 5.3. Structural and Interfacial Characterization

Structural characterization of composite films is mainly used to analyze interactions among different components, the micro-morphology of the film layer, and the structural order of the internal network [66]. Fourier transform infrared spectroscopy (FTIR) is often used to identify changes in functional groups and determine interfacial interactions such as hydrogen bonding and electrostatic interactions. X-ray diffraction (XRD) is mainly used to analyze crystalline structure and crystallinity changes. Scanning electron microscopy (SEM) can directly observe the uniformity, pore structure, and filler dispersion state of film surfaces and cross-sections [65]. If necessary, atomic force microscopy (AFM) and other methods can also be combined to further analyze interfacial details. For composite films, the significance of structural characterization is not only to describe film morphology, but more importantly to explain how component changes further affect mechanical, barrier, and functional properties [66].

### 5.4. Evaluation of Thermal Properties and Stability

Thermal-property evaluation mainly includes differential scanning calorimetry (DSC) and thermogravimetric analysis (TGA) [65]. DSC can be used to analyze glass transition temperature, melting behavior, and changes in chain-segment motion. TGA is used to determine the thermal decomposition characteristics and thermal stability range of film materials [66]. For composite films used in fruit and vegetable preservation, thermal evaluation does not directly correspond to shelf-life results as strongly as mechanical and barrier properties do. However, it can reflect whether the material structure becomes more stable after compounding and can provide a basis for selecting subsequent processing methods and analyzing storage adaptability.

### 5.5. Evaluation of Functional Activity and Preservation Effect

In addition to physicochemical properties, composite films also need to be evaluated for functional activity and actual preservation effect [67]. Functional activity evaluation usually includes antioxidant assays such as DPPH, ABTS, and FRAP, as well as antimicrobial assays such as inhibition-zone tests, colony counts, and minimum inhibitory concentration tests. If the film contains active compounds, release behavior and stability often also need to be measured [63]. Actual preservation evaluation usually uses the target fruit or vegetable as the experimental object and evaluates preservation performance based on indicators such as weight loss, firmness, color difference, soluble solids, titratable acidity, browning index, and microbial load [67]. For research on composite films for postharvest fruits and vegetables, the evaluation system becomes more complete only when material characterization results are connected with real preservation performance [68].

## 6. Mechanisms of Composite Films in Postharvest Preservation of Fruits and Vegetables

The effects of composite films in postharvest preservation of fruits and vegetables do not depend on a single property. Instead, they are achieved through multiple pathways, including regulation of the packaging microenvironment, slowing of physiological metabolism, inhibition of microbial invasion, and delay of oxidative damage [69]. Compared with traditional single-component film materials, composite films can integrate barrier effects, active-component delivery, and interfacial protection into one system, making them more conducive to delaying fruit and vegetable quality deterioration [70]. Overall, their preservation mechanisms mainly include gas regulation and metabolic inhibition, water retention and texture maintenance, antimicrobial and antiseptic effects, antioxidant activity and browning delay, and sustained release and synergistic action of active components [71].

### 6.1. Gas Regulation and Metabolic Inhibition

Fruits and vegetables continue to respire after harvest and continuously undergo O_2_ consumption, CO_2_ release, and metabolism related to ethylene regulation. Therefore, the ability of packaging materials to regulate gas exchange directly affects ripening and senescence processes [69]. Composite films can use their selective gas permeability to form a microenvironment with low O_2_ and moderately high CO_2_ inside the package, thereby reducing the oxidative decomposition of respiratory substrates and slowing metabolic rate. Some composite films also have ethylene adsorption or degradation ability, which can further weaken the accumulation of ripening signals and delay postharvest ripening [34]. In recent years, the development of photocatalytic composite films has shown that ethylene regulation by composite films has gradually expanded from passive barrier control to active degradation [70]. Therefore, the core role of composite films in gas regulation is to suppress respiratory metabolism, delay ripening, and maintain fruit firmness by reconstructing the packaging microenvironment [69].

### 6.2. Water Retention and Texture Maintenance

Postharvest water loss in fruits and vegetables is an important cause of shriveling, softening, and reduced marketability [71]. Composite films can maintain tissue water status by reducing water vapor transmission rate, reducing water loss caused by transpiration, and regulating humidity inside the package [72]. At the same time, an ideal composite film does not simply pursue extremely low moisture permeability. Instead, it establishes a dynamic balance between reducing water loss and suppressing condensation, so as to avoid promoting microbial proliferation due to excessive humidity [73]. On this basis, water retention is further expressed as texture maintenance, meaning that the decline in fruit and vegetable firmness is delayed by slowing decreases in cell turgor and tissue relaxation [69]. Therefore, water retention and texture maintenance are essentially different manifestations of the same regulatory process at different levels [71].

### 6.3. Antimicrobial and Antiseptic Effects

The surfaces of postharvest fruits and vegetables are rich in water and nutrients and are often accompanied by mechanical damage. They are therefore highly susceptible to bacterial and fungal invasion [74]. The antimicrobial and antiseptic effects of composite films do not rely on a single barrier effect. Instead, they are achieved through multiple pathways, including direct antimicrobial action by natural active components, controlled antimicrobial release from carrier structures, and broad-spectrum antimicrobial enhancement by functional components [75]. Essential oils, plant extracts, and polyphenols are the most common natural antimicrobial sources in composite films [76]. Encapsulation and sustained-release design help improve the stability and lasting activity of these active compounds, thereby enhancing the antiseptic effect [77]. In addition, metal-oxide nanoparticles and light-responsive semiconductor materials can further improve antimicrobial efficiency through reactive oxygen species generation and cell membrane damage [78]. Therefore, the essence of the antiseptic effect of composite films is to reduce decay rate and extend shelf life by continuously inhibiting the growth of pathogenic microorganisms [79].

### 6.4. Antioxidant Activity and Browning Delay

Oxidative damage and enzymatic browning are important causes of postharvest color deterioration and sensory quality decline in fruits and vegetables [80]. Natural polyphenols, essential oils, vitamins, and some functional nano-components in composite films can alleviate postharvest oxidative stress in fruits and vegetables by scavenging free radicals and reducing lipid peroxidation [81]. At the same time, composite films can inhibit the activity of browning-related enzymes such as polyphenol oxidase (PPO) and peroxidase (POD) to some extent, thereby reducing the conversion of phenolic substrates into quinones [82]. Some active films can also protect endogenous phenolic compounds and natural pigments in fruits and vegetables, further slowing appearance deterioration and nutrient loss [83]. Therefore, the regulation of antioxidant activity and browning delay by composite films is not limited to oxygen-barrier effects, but depends on the synergy among antioxidant action, enzyme inhibition, and substrate protection [84].

### 6.5. Sustained Release and Synergistic Action of Active Components

Sustained release and synergistic action of active components are important features that distinguish composite films from ordinary barrier films [85]. Many natural active compounds have good antimicrobial or antioxidant abilities, but they also have problems such as rapid volatilization, poor stability, and short action time. Therefore, structures such as microcapsules, nanoemulsions, metal–organic frameworks (MOFs), or porous carriers are needed to achieve stable encapsulation and sustained release [86]. This sustained-release effect helps prolong the effective action time of active components during storage and forms synergy with the barrier, antimicrobial, and antioxidant functions of the film itself. With the introduction of photothermal, enzyme-mimicking catalytic, and stimulus-responsive mechanisms, composite films have gradually developed from traditional passive barrier materials into functional packaging systems with multifunctional synergistic regulation [87]. This also indicates that the advantages of composite films in postharvest preservation of fruits and vegetables have expanded from basic protection to higher-level targeted regulation and multifunctional coupling [85].

For essential-oil-loaded composite films, release kinetics are especially important because the release rate determines both antimicrobial efficiency and sensory impact. Rapid release may produce a high local concentration and strong odor in the early storage period, whereas overly slow release may fail to reach the effective antimicrobial concentration. Therefore, controlled release should be designed not only to prolong antimicrobial action, but also to reduce sensory disturbance and improve consumer acceptance. Encapsulation, nanoemulsion delivery, and cyclodextrin inclusion are useful strategies for improving essential oil stability and regulating release behavior in active packaging systems [40,41,61].

### 6.6. Structure–Property–Mechanism Relationships and Multifunctional Synergy

The preservation mechanisms of composite films are not simply additive, but are determined by the structure–property–mechanism relationship of the whole film system. Gas regulation depends not only on total barrier performance, but also on gas selectivity. An ideal film should allow suitable O_2_ and CO_2_ exchange according to the respiration intensity of the target fruit or vegetable [63]. Excessive oxygen restriction may induce anaerobic respiration, while insufficient gas limitation may fail to suppress ripening [69,70].

The release behavior of active components is another key mechanistic factor. Effective preservation requires that release kinetics match the storage period, microbial growth pattern, and deterioration rate of the target produce. Otherwise, the film may show strong short-term activity but insufficient long-term protection, or may fail to reach the effective concentration required for microbial inhibition [88].

Matrix–bioactive interactions also influence preservation performance. Hydrogen bonding, electrostatic interaction, hydrophobic association, and encapsulation structures can affect the dispersion, stability, and release of active components [66]. These interactions further determine the mechanical strength, barrier properties, transparency, and biological activity of composite films. Moisture transport is also a dynamic process. A film with high moisture resistance can reduce weight loss, but excessive water retention may promote condensation and microbial growth [72,73]. Therefore, the final preservation effect depends on the dynamic balance among gas selectivity, moisture transport, active-component release, matrix—bioactive interactions, and produce-specific physiological responses [77,88].

In summary, the mechanism of composite films in postharvest preservation of fruits and vegetables can be summarized as follows: they slow metabolism and water loss by regulating gas exchange and water migration; they maintain quality stability through antimicrobial, antiseptic, antioxidant, and anti-browning effects; and they prolong the protective effect through the sustained release and synergistic action of active components [71]. In other words, the advantage of composite films does not lie in a single function, but in their ability to form multi-link synergistic regulation around the main postharvest deterioration processes of fruits and vegetables [79]. This mechanistic framework also provides a theoretical basis for analyzing specific applications in different fruit and vegetable systems in Section 7.

## 7. Application Progress of Composite Films in Postharvest Preservation of Fruits and Vegetables

**Figure 2:**
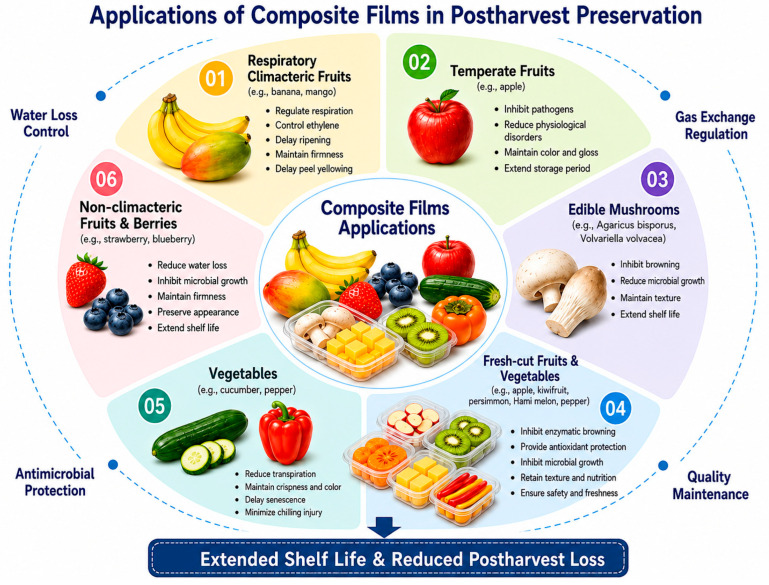
Main application types and key functional focuses of composite films in postharvest preservation of fruits and vegetables.

The application of composite films in postharvest preservation of fruits and vegetables has gradually shifted from early laboratory film-forming studies to validation in specific product scenarios. Existing studies show that the preservation effects of composite films vary significantly among different fruits and vegetables [22]. This difference mainly comes from differences in postharvest physiological activity, surface structure, water content, respiratory characteristics, and dominant deterioration mechanisms of fruits and vegetables [89]. Therefore, the focus of current composite film application research has gradually shifted from simply verifying preservation effects to analyzing the suitability of different composite film systems for different types of fruits and vegetables, and further clarifying the key quality indicators they improve and the degree of improvement. From the perspective of application objects, current studies mainly focus on four aspects: whole fruits, whole vegetables, fresh-cut fruits and vegetables, and comparison of preservation effects among different fruit and vegetable systems (Figure 2).

### 7.1. Preservation of Whole Fruits

Whole fruits are one of the most mature and intensively studied application objects for composite films. The main reason is that when the peel is intact, composite films or coatings can more easily form a continuous protective layer on the surface, thereby simultaneously regulating water migration, gas exchange, and microbial invasion. Recent reviews generally indicate that bio-based composite films and edible coatings have been widely used for postharvest preservation of whole fruits such as bananas, mangoes, strawberries, blueberries, cherries, grapes, apples, and kiwifruit [22]. They show comprehensive advantages such as delaying ripening, reducing weight loss, maintaining firmness, and reducing decay [89]. In climacteric fruits, the regulatory effect of composite films on ripening is especially obvious. Existing studies have shown that rice starch-based edible coatings can effectively delay ethylene biosynthesis and respiration rate in bananas, slow chlorophyll degradation, reduce weight loss, and better maintain pulp firmness during the early storage stage [90]. The shelf life of treated bananas can be extended to 12 days, whereas control fruits complete ripening within 7 days and quickly lose marketability. Similarly, composite coating systems also show potential in mango preservation by delaying ripening, reducing weight loss, maintaining firmness and titratable acidity, and slowing senescence-related quality deterioration.

This indicates that for climacteric fruits, the application value of composite films is first reflected in ripening delay and postharvest ripening control, rather than simple surface coating.

**Figure 3:**
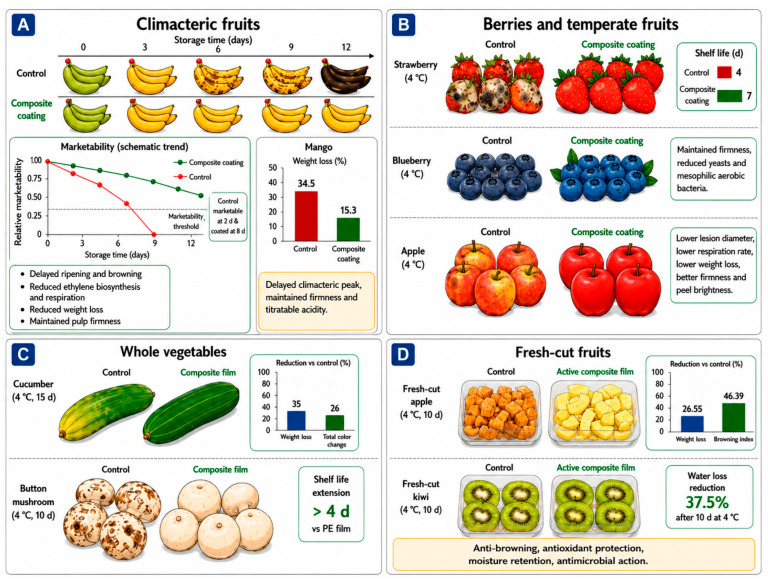
Typical applications of composite films/coatings in postharvest preservation of fruits and vegetables. (**A**) Climacteric fruits. Banana images show that composite coating delays ripening and browning, reduces ethylene biosynthesis, decreases weight loss, and maintains pulp firmness, extending marketability from 7 d to 12 d. Mango panel shows that composite coating reduces weight loss and helps maintain postharvest quality. (**B**) Berries and temperate fruits. Strawberry shelf life is extended to 7 d with composite coating. Blueberry coating maintains firmness and reduces microbial activity. Apple images show that chitosan/fennel seed composite film reduces lesion diameter, respiration, and weight loss. (**C**) Whole vegetables. Cucumber and mushroom images show the effects of composite films on weight loss and shelf-life extension. (**D**) Fresh-cut fruits. Composite films reduce browning and weight loss in fresh-cut apples and kiwifruit, with a 37.5% reduction in water loss after 10 d.

This pattern is summarized in Figure 3A. In this panel, banana is used to show the time-dependent difference between the control group and the composite coating group during storage. The control bananas rapidly turn yellow and then over-ripen, whereas coated bananas maintain better marketability for a longer period. The schematic marketability curve further indicates that the coating treatment slows the decline in commercial value. In the same panel, mango is used as another representative climacteric fruit, illustrating that composite coatings can reduce water loss and help maintain firmness and postharvest quality. Therefore, Figure 3A visually emphasizes that composite films are particularly useful for climacteric fruits because they can delay ripening, reduce ethylene-related quality deterioration, decrease weight loss, and maintain firmness (Figure 3A). For berries such as strawberries, blueberries, cherries, and grapes, the application focus of composite films is more reflected in reducing water loss, inhibiting mold growth, and maintaining fruit-surface integrity. Studies have shown that hyaluronic acid-based polysaccharide–protein composite edible coatings can improve changes in weight loss, pH, titratable acidity, and total soluble solids of strawberries during 15 days of storage [91], while also improving total phenol content, ascorbic acid retention, and DPPH scavenging ability. Another study showed that dissolution-resistant chitosan composite films could maintain structural stability under high humidity and extend the room-temperature shelf life of strawberries to 7 days [92]. In blueberry preservation, sodium alginate, pectin, and their composite coatings may not always significantly affect weight loss, but they can clearly improve fruit firmness and slow the growth of yeasts and mesophilic aerobic bacteria [93]. This indicates that the response of berry fruits to composite films is often reflected more in firmness maintenance, fruit-surface microbial control, and overall sensory maintenance, rather than only in delayed ripening.

In temperate fruits such as apples, composite films are more associated with pathogen inhibition, surface quality maintenance, and delayed color change. Studies have shown that chitosan/anise seed essential oil/octenyl succinic anhydride starch composite films have strong inhibitory effects against *Botrytis cinerea*, *Trichothecium roseum*, and *Penicillium expansum*. Among them, the F3 formulation has the best antifungal performance. When used for apple preservation, this film can reduce lesion diameter, reduce respiration rate and weight loss, and better maintain fruit firmness and peel brightness [94]. These results indicate that the application evaluation of whole apples usually focuses more on disease spread and appearance quality, rather than on water loss and mold growth as in berries. Figure 3B further compares the preservation effects of composite coatings in berries and temperate fruits. Strawberry is used to show the difference in shelf-life extension between the control and coating treatments, with the coated group maintaining better appearance and longer storage stability. Blueberry is presented to highlight the role of composite coatings in maintaining firmness and reducing yeasts and mesophilic aerobic bacteria. Apple is used as a representative temperate fruit, where the main effects of composite films are reflected in lower lesion diameter, reduced respiration rate and weight loss, and better maintenance of fruit firmness and peel brightness. Thus, Figure 3B shows that berries and temperate fruits respond to composite films in different ways: berries are more closely related to firmness maintenance and microbial control, whereas apples are more closely associated with pathogen inhibition and appearance preservation (Figure 3B). Overall, research on composite films for whole-fruit preservation is no longer limited to verifying usability. It is gradually developing toward targeted design based on the physiological characteristics of different fruits. For climacteric fruits such as bananas and mangoes, composite films place more emphasis on ripening delay and metabolic regulation. For berries such as strawberries, blueberries, grapes, and cherries, they place more emphasis on water retention, antiseptic protection, and maintenance of tissue integrity. For temperate fruits such as apples, disease prevention and appearance maintenance are more prominent. This indicates that the application of composite films in whole fruits has formed a relatively clear trend toward type-specific development [93].

### 7.2. Preservation of Whole Vegetables

Compared with whole fruits, the postharvest preservation goals of whole vegetables are not exactly the same. Most vegetables place more emphasis on water-loss control, maintenance of crisp texture, and preservation of surface color, rather than postharvest ripening delay as seen in some fruits. Therefore, the application effects of composite films in whole vegetables are often first reflected in reducing transpiration water loss, maintaining tissue firmness, and inhibiting surface discoloration. Review studies have pointed out that the application of biopolymer-based composite films and coatings in whole vegetables such as cucumbers, peppers, and edible fungi mainly depends on their integrated regulation of water migration, gas exchange, oxidation reactions, and pathogen growth [95]. This approach is also consistent with application summaries of chitosan and alginate systems [96]. In high-water-content vegetables such as cucumbers, the main application value of composite films is to reduce water loss, delay surface yellowing, and maintain firmness. Existing studies have shown that after 15 days of storage at 4 °C, thymol-based composite films can reduce the total weight loss rate of cucumbers by about 35% compared with the unpackaged group, reduce the total color difference by about 26%, and clearly slow the decline in firmness [86]. The authors suggest that this is mainly related to the good oxygen, carbon dioxide, and water vapor barrier properties of the film layer, which can reduce water loss and delay surface yellowing by inhibiting chlorophyll degradation. Thus, for high-water-content vegetables such as cucumbers, the preservation effect of composite films is not limited to the improvement of a single quality indicator. It is mainly reflected in comprehensive effects such as water retention, color stability, and texture maintenance.

For edible fungi and other whole fresh vegetables, the action focus of composite films is more inclined toward inhibiting browning, reducing weight loss, and controlling microbial proliferation. Studies have shown that cinnamaldehyde-tannic acid nanoemulsion/chitosan composite films have 100% ultraviolet-blocking ability, more than 99% antimicrobial effect, and strong DPPH and ABTS free-radical scavenging abilities [97]. When used for *Agaricus bisporus* preservation, they can extend the preservation period by more than 4 days compared with PE films. Another study reported that polysaccharide composite films impregnated with ultrasound-assisted nanoemulsions could significantly reduce PPO activity and browning index during storage of *Agaricus bisporus* and *Volvariella volvacea* [98], while also reducing weight loss and firmness decline. This indicates that the response of edible fungi to composite films is not only reflected in water-loss control, but also depends more on the combined effects of composite films in water retention, enzyme inhibition, antioxidant activity, and antimicrobial action. From a broader perspective of whole-vegetable applications, composite films have gradually developed from simple barrier packaging to active and functional packaging. Review studies indicate that after polyphenols, essential oils, or nanofillers are introduced into polysaccharide or protein matrices, the moisture-barrier properties, antioxidant activity, and antimicrobial activity of films can usually be enhanced simultaneously [99]. These functions are exactly the core requirements for maintaining postharvest vegetable quality. Therefore, the application of composite films in whole vegetables is no longer merely a strategy to replace traditional plastic films, but a more targeted material-design approach based on the dominant deterioration pathways of different vegetables [95]. The representative effects in whole vegetables are shown in Figure 3C. Cucumber is used as a high-water-content vegetable to show that composite films can reduce weight loss and total color difference during cold storage. This result is consistent with the main preservation demand of cucumbers, namely reducing water loss, delaying yellowing, and maintaining firmness. Button mushroom is used as a representative edible fungus. Compared with the control group, the composite film group shows better appearance quality and a shelf-life extension of more than 4 days compared with PE film. Therefore, Figure 3C indicates that the preservation value of composite films in whole vegetables is mainly reflected in water retention, color stability, texture maintenance, browning inhibition, and microbial control (Figure 3C).

### 7.3. Preservation of Fresh-Cut Fruits and Vegetables

Fresh-cut fruits and vegetables represent the most complex application scenario for composite films. Compared with whole fruits and vegetables, fresh-cut processing breaks the natural surface barrier, increases exposed tissue area, and significantly accelerates respiratory metabolism, water loss, enzymatic browning, and microbial contamination [100]. Therefore, fresh-cut products usually have higher functional requirements for composite films than whole products and rely more on the synergistic effects of the film layer in moisture barrier, antioxidant activity, anti-browning action, and antimicrobial activity. This trend is also consistent with conclusions from studies on starch-based active films [101]. Fresh-cut apples are currently one of the most intensively studied model systems. Existing studies have shown that sodium alginate-carnauba wax-calcium ascorbate composite films perform best when the calcium ascorbate addition level is 0.4%. Their water vapor transmission rate can be as low as 0.65 g·mm/(cm^2^·d·kPa), and they can better maintain the color, firmness, titratable acidity, and soluble solids of fresh-cut apples, while inhibiting increases in PPO activity and total colony count [102]. Whey protein-based emulsion composite coatings can reduce the weight loss rate and browning index of fresh-cut apples by 26.55% and 46.39%, respectively [103]. They delay quality deterioration by inhibiting respiration rate, changes in PPO activity, and the accumulation of H_2_O_2_ and MDA. In addition, soy protein isolate/*Artemisia sphaerocephala* Krasch. gum composite films loaded with pomegranate peel extract have good moisture-barrier, oxygen-barrier, and antioxidant properties. The cumulative release rate of polyphenols is still only 63.83% at day 7, indicating stable sustained-release characteristics. They can also effectively reduce browning index and weight loss in fresh-cut apples. These results show that the response of fresh-cut apples to composite films is mainly reflected in the synergistic effects of anti-browning action, antioxidant activity, and water retention. Figure 3D illustrates the application of active composite films in fresh-cut fruits. Fresh-cut apple is used to show the reduction in weight loss and browning index after film treatment, indicating that composite films can effectively inhibit enzymatic browning and water loss after tissue cutting. Fresh-cut kiwifruit is used to show the water-loss reduction effect, with the treated group showing a 37.5% decrease in water loss after 10 days of storage at 4 °C. These examples demonstrate that fresh-cut fruits have stronger requirements for composite films than whole products because cutting destroys the natural surface barrier and accelerates moisture loss, oxidation, browning, and microbial contamination. Therefore, Figure 3D highlights the multifunctional value of composite films in fresh-cut systems, including anti-browning, antioxidant protection, moisture retention, and antimicrobial action (Figure 3D).

In addition to apples, fresh-cut kiwifruit, fresh-cut persimmon, and fresh-cut Hami melon systems also show strong compatibility with composite films. Studies have shown that coffee-ground extract-enhanced chitosan-sodium alginate composite films can reduce water loss in fresh-cut kiwifruit by 37.5% during 10 days of storage at 4 °C, while delaying decay and quality decline [104]. Apple pectin-based composite coatings combined with citric acid, calcium chloride, and antimicrobial agents can effectively inhibit browning and microbial growth in fresh-cut ‘Rojo Brillante’ persimmon, maintaining a marketable period of about 7 days at 5 °C [105]. Another study reported that *Flammulina velutipes* polysaccharide/sodium carboxymethyl cellulose composite films can significantly delay weight loss, softening, nutrient loss, and total colony growth in fresh-cut Hami melon [106]. This indicates that in fresh-cut fruit systems, the main value of composite films still lies in the comprehensive inhibition of water loss, browning, oxidation, and microbial contamination, which are amplified after tissue damage. In fresh-cut vegetables, the application of composite films further emphasizes both functionality and display quality. Studies have shown that gellan gum/bacterial cellulose/nano-TiO_2_-CuO functional films can effectively inhibit softening, reddening, browning, and decay of fresh-cut peppers during storage. They also have good anti-fogging, bendable, and thermoreversible properties. Representative applications of composite films/coatings in different fruit and vegetable systems are summarized in Table 2. Overall, the application of composite films in fresh-cut fruits and vegetables has developed from simple surface coating into multifunctional packaging systems, and fresh-cut systems best reflect the advantages of multi-component synergy in composite films [107].

### 7.4. Comparison of Preservation Effects Among Different Fruit and Vegetable Types

Different types of fruits and vegetables differ significantly in postharvest physiological activity, surface structure, water content, respiration intensity, and dominant deterioration mechanisms. Therefore, the preservation effects of composite films also show clear type dependence. Existing studies generally indicate that there is no single optimal composite film formulation that can be applied to all fruits and vegetables and achieve the same preservation effect. Instead, targeted design is needed according to the main deterioration problems of different fruits and vegetables. Overall, climacteric fruits rely more on gas regulation and ripening delay [68]. Non-climacteric fruits and berries rely more on prevention of water loss, mold growth, and appearance deterioration, while fresh-cut fruits and vegetables have higher requirements for anti-browning, antioxidant, and antimicrobial functions [107].

The response of climacteric fruits to composite films is usually first reflected in delayed ripening and respiration inhibition. Jafarzadeh et al. [95] pointed out that these fruits can continue to ripen after harvest and are accompanied by higher ethylene production and respiration rates. Therefore, they are more sensitive to the oxygen, water vapor, and ethylene regulation ability of packaging materials. Mao et al. [99], in summarizing applications of starch-based active films in bananas and mangoes, also pointed out that tannic acid-modified corn starch/chitosan composite films can extend banana shelf life from 3 days to 6 days and reduce weight loss by 14%. In mangoes, they mainly slow peel yellowing and keep the fruit relatively fresh after 11 days. By contrast, non-climacteric fruits and berries do not necessarily show significant delayed postharvest ripening, but they often show more obvious advantages of composite films in weight-loss control, fruit-surface firmness maintenance, and microbial inhibition. Blueberry studies show that although sodium alginate and pectin coatings may not always significantly reduce weight loss, they have good effects on firmness maintenance and microbial control. Thick-skinned fruits such as pomegranates are more suitable for improving antiseptic ability by introducing natural essential oils and plant extracts. Related studies have shown that sodium alginate- and chitosan-based coatings containing lemongrass essential oil as the active component can completely inhibit the spore germination of pomegranate decay fungi and significantly reduce fruit decay [108]. Therefore, for non-climacteric fruits and berries, composite films are more often reflected as comprehensive protective materials that reduce water loss, inhibit disease development, and maintain appearance quality. Compared with fruits, whole vegetables have different evaluation priorities. Vegetables usually place more emphasis on water retention, crispness, and color maintenance [95]. Therefore, the value of composite films in cucumbers, peppers, edible fungi, and other systems is more reflected in reduced weight loss, reduced color difference, maintained firmness, and maintained surface condition, rather than postharvest ripening delay. Fresh-cut fruits and vegetables have tissue damage that amplifies browning, oxidation, and microbial contamination. Therefore, they depend most on the synergistic protection of antioxidant, anti-browning, and antimicrobial functions [100]. For this reason, fresh-cut systems often best reflect the advantages of multifunctional integration in composite films [103,107].

From an application perspective, differences among fruit and vegetable types essentially reflect differences in dominant deterioration mechanisms. Material selection and functional configuration of composite films cannot simply follow a universal formulation, but should be driven by the physiological characteristics, surface structure, and main failure pathways of the target product [95]. Existing studies have clearly shown that evaluation of composite films should not use shelf-life extension alone as the judgment criterion. It should also combine the most important quality indicators of different fruits and vegetables, such as the postharvest ripening speed of bananas, the decay rate of berries, the color and firmness of cucumbers, and the browning index and antioxidant status of fresh-cut apples. Only by establishing the correspondence among fruit and vegetable type, dominant deterioration mechanism, functional demand, and formulation design can composite film technology truly move from general material research toward precision application [104]. In summary, the application of composite films in postharvest preservation of fruits and vegetables is gradually shifting from general verification of preservation effectiveness toward demand-oriented targeted design. The dominant deterioration mechanisms of different fruits and vegetables determine the functional focus of composite films. Among them, climacteric fruits place more emphasis on regulation of the ripening process; berries and whole vegetables pay more attention to water retention and anti-mold effects; and fresh-cut fruits and vegetables need the combined functions of anti-browning, antioxidant, and antimicrobial effects. This trend suggests that future application studies should more closely connect product characteristics with the functional configuration of films.

## 8. Current Challenges and Future Perspectives

### 8.1. Produce-Specific Design and Application Boundaries

**Figure 4:**
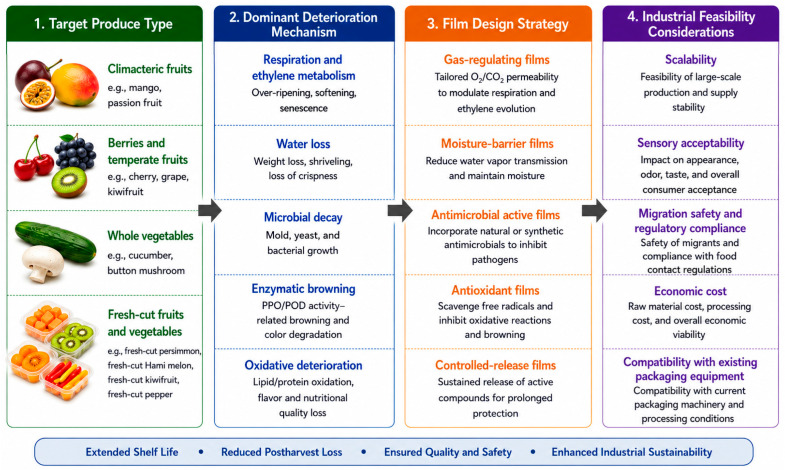
Produce-specific design logic and practical feasibility framework for composite films in postharvest preservation of fruits and vegetables. The figure summarizes the relationships among target produce type, dominant deterioration mechanism, film design strategy, and industrial feasibility. It highlights that composite film design should be guided by produce-specific deterioration patterns and should also consider scalability, sensory acceptability, migration safety, economic cost, and compatibility with existing packaging equipment.

Although composite films have shown great potential in postharvest preservation of fruits and vegetables, their effectiveness should not be understood as universally applicable to all produce. Different fruits and vegetables have distinct dominant deterioration pathways, including respiration and ethylene-related ripening, water loss, enzymatic browning, microbial decay, texture softening, and nutrient degradation. Therefore, the design of composite films should shift from a material-driven approach to a produce-specific and demand-oriented strategy. For climacteric fruits, gas regulation and ethylene-related ripening control should be emphasized. For berries, surface integrity, fungal inhibition, and water retention are more important. For whole vegetables, moisture regulation, color stability, and texture maintenance are usually the main concerns. For fresh-cut products, anti-browning, antioxidant protection, and microbial control become more critical because the natural surface barrier has been destroyed. Future studies should further clarify the matching relationship among produce type, dominant deterioration mechanism, film structure, and functional component design [104]. Based on these produce-specific deterioration patterns, the design of composite films should be further connected with functional formulation strategies and practical feasibility considerations. A general design logic and feasibility map is summarized in Figure 4.

### 8.2. Standardization and Comparability of Evaluation Methods

Another important challenge is the limited comparability among different studies. Storage temperature, relative humidity, film thickness, coating method, produce maturity, cultivar, storage duration, and quality indicators vary greatly across studies. As a result, it is difficult to directly compare the preservation efficiency of different composite film systems. For example, shelf-life extension under low-temperature storage cannot be directly compared with that under room-temperature storage. Similarly, a film that effectively reduces weight loss may not necessarily inhibit browning or microbial growth. Therefore, future studies should report key experimental parameters more systematically, including film thickness, gas permeability, water vapor transmission rate, active-component loading level, release behavior, coating method, storage conditions, and initial maturity of the produce. Standardized evaluation systems will help improve the reliability and practical value of comparative studies.

### 8.3. Safety, Sensory Quality, and Regulatory Concerns

Safety and sensory quality are key factors that determine whether composite films can move from laboratory research to practical application. For nanocomposite films, nanoparticle migration, long-term exposure risks, cytotoxicity, environmental release, and food-contact regulatory requirements should be carefully evaluated. Although nanomaterials can improve barrier, mechanical, antimicrobial, and controlled-release properties, their application should not be assessed only by preservation performance. Migration testing, toxicological assessment, dietary exposure evaluation, and environmental risk analysis are also necessary [42,43,64].

For essential-oil-loaded composite films, sensory quality and consumer acceptance should be treated as key practical evaluation criteria rather than secondary observations. Although essential oils can provide antimicrobial and antioxidant effects, their strong characteristic aromas may interfere with the natural flavor and freshness perception of fruits and vegetables when used at high concentrations [40,41]. This issue is particularly important for fresh fruits, fresh-cut products, and minimally processed vegetables, because these products are usually consumed directly and their market value is closely related to natural aroma, taste, color, and freshness perception. Therefore, future studies should evaluate essential-oil-loaded composite films not only by microbial inhibition, antioxidant activity, and shelf-life extension, but also by sensory analysis, volatile-compound profiling, and consumer acceptance tests [40,41]. In addition, researchers should report the loading level of essential oils, the sensory evaluation method, and the acceptable concentration range more clearly, so that the preservation effect can be balanced with product flavor and market acceptability.

### 8.4. Structure–Property–Preservation Relationships and Release Behavior

The relationship among film structure, material properties, and real preservation performance remains insufficiently clarified. Improved tensile strength, reduced water vapor transmission rate, or enhanced antimicrobial activity does not always directly lead to better shelf-life extension. The final preservation effect depends on whether these material properties match the physiological characteristics and deterioration pathways of the target produce. Therefore, future studies should strengthen the connection among microscopic structure, interfacial interactions, gas and water transfer behavior, active-component release, and quality changes during storage.

Release behavior is especially important for active composite films. Rapid release of antimicrobial or antioxidant components may provide strong short-term activity, but it may also cause sensory changes and insufficient long-term protection. In contrast, overly slow release may fail to reach the effective concentration required for microbial inhibition or oxidative protection. Therefore, release kinetics should be designed according to the storage period, microbial growth pattern, respiration intensity, and deterioration rate of the target fruit or vegetable [61].

### 8.5. Industrial Feasibility and Commercial Application

Although many composite films show good preservation effects under laboratory conditions, their industrial application still faces several practical challenges. First, large-scale manufacturing requires stable raw-material supply, controllable film-forming processes, uniform thickness, reproducible mechanical properties, and compatibility with existing packaging equipment. Many bio-based or active composite films are still prepared by casting or laboratory coating methods, which are difficult to directly translate into high-throughput industrial production [109,110].

Second, composite films must compete with conventional low-cost plastic packaging materials, such as PE, PP, and PVC, which already have mature processing systems, stable barrier properties, and low production costs [109]. Therefore, the commercial value of composite films should not be evaluated only by shelf-life extension, but also by cost, processing efficiency, mechanical reliability, storage stability, biodegradability, and regulatory compliance.

Third, commercial postharvest circulation involves temperature fluctuations, transport vibration, mechanical damage, humidity changes, microbial contamination, and long-term storage. A film that performs well under controlled laboratory conditions may not show the same stability during transportation and commercialization. Therefore, future studies should move from small-scale laboratory validation to pilot-scale and industrial-scale testing, and should evaluate packaging compatibility, transportation stability, consumer acceptance, and life-cycle environmental impact [109,110].

### 8.6. Smart, Responsive, and AI-Assisted Composite Films

Future development of composite films should move toward biodegradable, multifunctional, and intelligent packaging platforms. Colorimetric freshness indicators are one promising direction. Natural pigments such as anthocyanins, curcumin, and betalains can respond to pH changes, volatile amines, or microbial metabolites, thereby providing visual information on freshness changes during storage [61,111]. Such systems may help consumers and retailers monitor fruit and vegetable quality more directly.

Stimuli-responsive release systems are another important direction. Active components can be designed to respond to humidity, pH, temperature, enzymes, light, or microbial metabolites, so that antimicrobial or antioxidant agents are released when deterioration risk increases. This on-demand release strategy may improve active-compound utilization efficiency and reduce unnecessary sensory impact [88].

AI-assisted material optimization may also provide new opportunities for composite film design. Machine learning models can be used to analyze relationships among material composition, processing parameters, film properties, release behavior, and preservation outcomes. This may help screen suitable substrate combinations, predict barrier and mechanical properties, and optimize active-component loading more efficiently. In the long term, biodegradable multifunctional smart packaging platforms should integrate safety, sustainability, intelligent monitoring, controlled release, and industrial feasibility [111].

## 9. Conclusions

Composite films provide a promising strategy for postharvest preservation of fruits and vegetables because they can integrate barrier protection, mechanical support, antimicrobial activity, antioxidant function, and active-component release within one material system. Compared with traditional single-component films, their main advantage lies in multi-component coordination and multifunctional regulation. They can slow respiratory metabolism and water loss by regulating the transmission of O_2_, CO_2_, and water vapor, and they can also maintain quality stability through antimicrobial, antiseptic, antioxidant, anti-browning, and sustained-release effects. Therefore, composite films show clear potential for maintaining the firmness, color, nutritional quality, and sensory quality of fruits and vegetables.

From the perspective of material systems, polysaccharides, proteins, and lipids remain the main substrates for composite film construction. Polysaccharides mainly provide film-forming ability and basic barrier properties, proteins are more conducive to forming flexible and uniform film networks, and lipids are useful for improving hydrophobicity and water-loss prevention. Meanwhile, functional components such as phenolic compounds, essential oils, natural pigments, nanofillers, and controlled-release carriers have gradually transformed composite films from simple physical barriers into active and responsive packaging systems. However, the preservation performance of composite films should not be understood as the simple accumulation of multiple functions. Their effectiveness depends on the matching relationship among film structure, mass-transfer properties, active-component behavior, storage conditions, and the dominant deterioration mechanisms of specific fruits and vegetables.

From the application perspective, different fruits and vegetables differ greatly in postharvest physiological activity, surface structure, water content, respiratory characteristics, and dominant deterioration pathways. Therefore, there is no universal composite film formulation suitable for all products. Climacteric fruits require more attention to gas regulation and ripening delay; berries and non-climacteric fruits require more emphasis on water-loss control, fungal inhibition, and appearance maintenance; whole vegetables depend more on moisture regulation and texture preservation; and fresh-cut fruits and vegetables require stronger anti-browning, antioxidant, and antimicrobial functions. This indicates that composite film research has shifted from simply verifying preservation effectiveness to exploring the matching relationship among produce type, deterioration mechanism, functional demand, and formulation design.

Although current studies have demonstrated considerable progress, several issues remain unresolved. These include insufficient produce-specific design, limited comparability among studies, unclear structure–property–preservation relationships, uncertain release behavior of active components, potential safety and sensory concerns, and insufficient industrial-scale validation. Future research should shift from general performance improvement to precision design based on fruit and vegetable type, deterioration pathway, storage condition, and real application scenario. In addition, intelligent indicators, stimuli-responsive release systems, AI-assisted material optimization, and biodegradable multifunctional platforms may become important directions for future composite film development. Only by improving material safety, structural stability, functional durability, and application-scenario adaptability can composite films better serve postharvest loss reduction and green packaging development for fruits and vegetables.

## Figures and Tables

**Table 1:** Comparison of main substrate types and functional characteristics of composite films.

Substrate Category	Representative Materials	Core Functional Characteristics	Main Advantages	Main Disadvantages	Typical Applications in Composite Films	References
Polysaccharides	Chitosan, Cellulose, Alginate, Pectin	Good film-forming properties, basic barrier properties, some materials have natural antimicrobial activity	Widely available, biodegradable, good safety profile, suitable as the main matrix for preservation films	Poor water resistance and mechanical performance when used alone	Used as the main substrate, providing the basic film-forming framework, gas barrier, and active-component loading platform	[52]
Cellulose-derived Reinforcing Materials	Cellulose Nanocrystals, Nanocellulose	Strong structural reinforcement ability, improves thermal stability, mechanical properties, and UV resistance	Reduces water vapor permeability, enhances overall physicochemical properties, and extends shelf life	Typically not used as the primary film-forming substrate but rather as a reinforcing phase	Used to enhance the structural stability, moisture barrier, and overall performance of films	[52,53]
Proteins	Gelatin, Whey Protein, Soy Protein	Good film-forming properties and transparency, easy to form uniform films, good carrier for active components	Improves gas-barrier properties, mechanical strength, and water resistance, beneficial for delaying ripening and oxidative changes	Poor moisture resistance compared to lipid-based systems	Used to form flexible and uniform film networks, and as a carrier for antioxidants and antimicrobial agents	[53,54]
Lipids	Plant oils, Waxes, Fatty acids	Strong hydrophobicity, excellent moisture-barrier performance properties, reduces water loss and migration of volatile compounds	Effective in delaying water loss and maintaining shelf life, can also act as a carrier for essential oils and nanoparticles	Weak mechanical performance when used alone	Used to improve hydrophobicity and moisture resistance, and combined with polysaccharides or proteins in composite systems	[55]

**Table 2:** Comparative summary of composite films/coatings for postharvest preservation of representative fruits and vegetables.

Target Fruit/Vegetable	Composite Film/Coating System	Dominant Preservation Mechanism	Shelf-Life Extension/Major Outcome	Major Limitation	Industrial Feasibility	References
Banana	Starch-based coating; tannic acid-modified corn starch/chitosan composite film	Regulation of respiration and ethylene-related ripening; delayed peel yellowing; reduced water loss	Marketability was extended from 7 d to 12 d in starch-based coating systems; tannic acid-modified corn starch/chitosan film extended shelf life from 3 d to 6 d and reduced weight loss by about 14%	Preservation effect is strongly affected by maturity stage, coating uniformity, storage temperature, and gas-permeability balance	Medium. Starch and chitosan are accessible and food-compatible, but coating uniformity and scalable processing still need optimization	[90,99]
Mango	Tannic acid-modified corn starch/chitosan composite film	Delayed respiratory climacteric; regulation of ripening metabolism; maintenance of firmness and titratable acidity	The film slowed peel yellowing, reduced weight loss, and maintained freshness during storage	Effect depends on cultivar, maturity stage, coating thickness, and storage temperature; excessive gas restriction may affect flavor	Medium. Natural polymer coatings are relatively practical, but large-scale coating control and sensory effects need further validation	[99]
Strawberry	Chitosan-based film/coating; polysaccharide–protein composite edible coating	Antimicrobial and antioxidant protection; moisture retention; maintenance of firmness and bioactive compounds	Composite coatings extended strawberry shelf life to about 7–8 d and improved retention of total phenols, ascorbic acid, and antioxidant capacity	Strawberry has a fragile surface; coating thickness, gloss, flavor, and active-agent safety require careful control	Medium to low. Preservation effect is clear, but sensory acceptability and safety of active components are key barriers	[91,92]
Blueberry	Sodium alginate coating; pectin coating; caseinate-carboxymethyl chitosan composite coating	Water-barrier effect; firmness maintenance; microbial inhibition; hydrophobic modification by lipid components	Some coatings did not always significantly reduce weight loss, but they maintained firmness and slowed yeast and mesophilic aerobic bacterial growth; caseinate-carboxymethyl chitosan coating reduced weight loss and inhibited yeast/mold growth	Waxy fruit surface may limit coating adhesion; effect on weight loss may be inconsistent; opacity and sensory changes should be considered	Medium. Coating-based preservation is feasible, but stable adhesion and appearance control are required	[93]
Grape, Cherry	Bio-based edible coating or composite film	Moisture retention; antifungal protection; maintenance of surface integrity and sensory quality	Composite coatings mainly reduced weight loss, inhibited decay, and maintained firmness and appearance quality	Surface bloom layer, gloss, and sensory quality may be affected; cultivar-specific optimization is needed	Medium. Edible coatings are feasible, but formulation should be adjusted according to fruit-surface characteristics	[75]
Pomegranate	Sodium alginate/chitosan active coating containing lemongrass essential oil	Antifungal activity; inhibition of fungal spore germination; reduction of disease development	Lemongrass essential oil coating completely inhibited spore germination of decay fungi and significantly reduced fruit decay	Essential oil odor, concentration control, and release behavior may affect sensory acceptance	Medium. Thick-skinned fruits are relatively suitable for essential oil-based coatings, but sensory and regulatory evaluation is needed	[99]
Apple (Whole)	Chitosan/fennel seed essential oil/starch sodium octenyl succinate composite film	Antifungal activity; reduced respiration; maintenance of firmness and peel brightness	Composite film reduced lesion diameter, respiration rate, and weight loss, while maintaining fruit firmness and peel brightness	Essential oil odor and release rate may affect fruit aroma; film transparency and sensory acceptance should be considered	Medium. The system is promising, but flavor compatibility and large-scale application require further validation	[94]
Cucumber	Cinnamaldehyde-based composite film	Water retention; delayed yellowing; maintenance of firmness and color stability	Total weight loss was reduced by about 35%, total color difference was reduced by about 26%, and firmness decline was slowed	High water content makes cucumber sensitive to humidity imbalance and condensation; excessive barrier may promote microbial growth	Medium. Application is feasible, but water vapor transmission and anti-condensation control are important	[86]
Edible Mushrooms (Button Mushroom, Straw Mushroom)	Cinnamaldehyde-tannic acid nanoemulsion/chitosan composite film; ultrasound-assisted nanoemulsion-impregnated polysaccharide composite film	Moisture retention; PPO inhibition; browning reduction; antimicrobial and antioxidant protection	Button mushroom shelf life was extended by more than 4 d compared with PE film; composite films also reduced PPO activity, browning index, weight loss, and firmness decline in edible mushrooms	Mushrooms are highly sensitive to humidity and browning; excessive moisture retention may cause condensation and microbial growth	Medium. Good application potential, but packaging humidity control and anti-browning stability are critical	[97,98]
Fresh-cut Apple	Sodium alginate-carnauba wax-calcium ascorbate composite film; whey protein-based emulsion coating; soy protein isolate/*Artemisia sphaerocephala* Krasch. gum composite film containing pomegranate peel extract	Anti-browning protection; antioxidant activity; moisture retention; microbial inhibition; sustained release of active compounds	Sodium alginate-carnauba wax-calcium ascorbate film maintained color, firmness, titratable acidity, and soluble solids; whey protein coating reduced weight loss and browning index by 26.55% and 46.39%, respectively; pomegranate peel extract film reduced browning and weight loss	Fresh-cut apples are highly sensitive to browning and sensory changes; active-compound concentration and release rate need precise control	Medium. Fresh-cut apple is a strong model system, but commercial use requires sensory validation and cold-chain compatibility	[102,103]
Fresh-cut Kiwifruit	Chitosan-sodium alginate composite film enhanced with spent coffee-ground extract	Moisture retention; antioxidant protection; antimicrobial support	Water loss was reduced by 37.5% during 10 d of storage at 4 °C, while decay and quality deterioration were delayed	Preservation depends on cold-chain storage; extract color, flavor, and batch-to-batch composition should be evaluated	Medium. Bioactive extract use is promising, but standardization of extract composition is needed	[104]
Fresh-cut Persimmon	Apple pectin-based composite coating containing citric acid, calcium chloride, and antimicrobial agents	Browning inhibition; microbial control; firmness maintenance through calcium-assisted texture protection	Fresh-cut ‘Rojo Brillante’ persimmon maintained a marketable period of about 7 d at 5 °C	Performance depends on cultivar, cut surface condition, and cold storage; formulation may affect flavor	Medium. Practical for fresh-cut fruit systems, but cold-chain support and sensory testing are required	[105]
Fresh-cut Hami Melon	*Flammulina velutipes* polysaccharide/sodium carboxymethyl cellulose composite film	Water retention; texture maintenance; nutrient protection; microbial inhibition	Composite film significantly delayed weight loss, softening, nutrient loss, and total colony growth	High moisture and sugar content increase microbial risk; packaging condensation should be controlled	Medium. Suitable for fresh-cut melon, but microbial safety and cold-chain management are essential	[106]
Fresh-cut Pepper	Gellan gum/bacterial cellulose/nano-TiO_2_-CuO functional film	Antimicrobial activity; anti-browning effect; anti-fogging function; physical protection	Functional film inhibited softening, reddening, browning, and decay, and showed anti-fogging, bendable, and thermoreversible properties	Nanomaterial migration, regulatory safety, and consumer acceptance need further evaluation	Low to medium. Functional performance is strong, but nanomaterial safety and regulatory compliance are key barriers	[25]

## Data Availability

No new data were created or analyzed in this study. Data sharing is not applicable to this article.
